# Medicine availability and affordability for paediatric cancers, China

**DOI:** 10.2471/BLT.24.291640

**Published:** 2024-11-12

**Authors:** Lin Bai, Tao Huang, Huangqianyu Li, Luwen Shi, Avram Denburg, Sumit Gupta, Xiaodong Guan

**Affiliations:** a Department of Pharmacy Administration and Clinical Pharmacy, School of Pharmaceutical Sciences, Peking University, 38 Xueyuan Road, Haidian District, Beijing, 100191, China.; b Division of Haematology/Oncology, The Hospital for Sick Children, Toronto, Canada.

## Abstract

**Objective:**

To investigate access to essential anticancer medicines for children throughout China.

**Methods:**

We obtained cross-sectional drug use data for 2021 from 55 tertiary children's hospitals in seven geographical regions (one third of public children's hospitals in mainland China). Affordability was assessed by comparing the single-day copayment for each medicine with the same generic name and route of administration (i.e. product) or for a treatment course with daily disposable income per capita in each region. The median availability and affordability of all 33 anticancer medicines in the 2021 *WHO Model list of essential medicines for children* were calculated and compared by region and medicine type.

**Findings:**

Although all medicines had been approved in China, 14 (42.4%) were available in under 50% of hospitals and six (18.2%) products had a median single-day copayment exceeding daily disposable income. Median availability was higher among the 19 medicines with approval for paediatric indications than among the 14 without (80.0% versus 48.2%, respectively; *P* < 0.001). Overall, 42.4% (14/33) of medicines had both good availability and affordability; the lowest proportion was in north-west China (30.3%, 10/33). A Chinese resident needed to work for 5.3 days to afford 4 weeks’ induction therapy for acute lymphoblastic leukaemia, the most common childhood cancer.

**Conclusion:**

Access to essential anticancer medicines for children remained suboptimal in China and varied across regions. Fewer than half the medicines studied had both good availability and affordability. Actions are warranted to address potential shortages and decrease the financial burden on families.

## Résumé

**Objectif:**

Étudier l’accès aux médicaments anticancéreux essentiels pour les enfants dans l’ensemble de la Chine.

**Méthodes:**

Nous avons obtenu des données transversales sur l’utilisation des médicaments en 2021 auprès de 55 hôpitaux pédiatriques tertiaires dans sept régions géographiques (un tiers des hôpitaux pédiatriques publics en Chine continentale). L’accessibilité financière a été évaluée en comparant d’une part le ticket modérateur d’une journée pour chaque médicament ayant le même nom générique et la même voie d’administration (c’est-à-dire un produit) ou pour une série de traitements et d’autre part le revenu disponible quotidien par habitant dans chaque région. La disponibilité médiane et l'accessibilité financière des 33 médicaments anticancéreux figurant sur la *Liste modèle de l’OMS des médicaments essentiels destinés à l’enfant* ont été calculées et comparées par région et par type de médicament.

**Résultats:**

Bien que tous les médicaments aient été approuvés en Chine, 14 (42,4%) étaient disponibles dans moins de 50% des hôpitaux et six produits (18,2%) étaient associés à un ticket modérateur médian quotidien qui dépassait le revenu disponible quotidien. La disponibilité médiane était plus élevée parmi les 19 médicaments approuvés pour des indications pédiatriques que parmi les 14 médicaments non approuvés (80,0% contre 48,2%, respectivement; *P* < 0,001). Dans l’ensemble, 42,4% (14/33) des médicaments étaient à la fois disponibles et abordables; c’est dans le nord-ouest de la Chine que la proportion était la plus faible (30,3%, 10/33). Un résident chinois devait travailler 5,3 jours pour s’offrir un traitement d’induction de 4 semaines contre la leucémie lymphoblastique aiguë, le cancer le plus fréquent chez l’enfant.

**Conclusion:**

L’accès aux médicaments anticancéreux essentiels pour les enfants reste perfectible en Chine et varie d’une région à l’autre. Moins de la moitié des médicaments étudiés étaient à la fois disponibles et abordables. Des actions sont justifiées pour remédier aux pénuries potentielles et réduire la charge financière qui pèse sur les familles.

## Resumen

**Objetivo:**

Investigar el acceso de los niños de toda China a los medicamentos esenciales contra el cáncer.

**Métodos:**

Se obtuvieron datos transversales de consumo de fármacos para 2021 de 55 hospitales infantiles terciarios de siete regiones geográficas (un tercio de los hospitales infantiles públicos de China continental). La asequibilidad se evaluó al comparar el copago de un día por cada medicamento con el mismo nombre genérico y vía de administración (es decir, producto) o por un ciclo de tratamiento con los ingresos diarios disponibles per cápita en cada región. Se calculó la mediana de disponibilidad y asequibilidad de los 33 medicamentos contra el cáncer incluidos en la *Lista Modelo OMS 2021 de medicamentos pediátricos esenciales* y se comparó por región y tipo de medicamento.

**Resultados:**

Aunque todos los medicamentos habían sido aprobados en China, 14 (42,4%) estaban disponibles en menos del 50% de los hospitales y seis (18,2%) productos tenían un copago medio de un solo día superior a los ingresos diarios disponibles. La mediana de disponibilidad fue mayor entre los 19 medicamentos con aprobación para indicaciones pediátricas que entre los 14 sin ella (80,0% frente a 48,2%, respectivamente; *p* < 0,001). En general, el 42,4% (14/33) de los medicamentos tenían buena disponibilidad y asequibilidad; el porcentaje más bajo se registró en el noroeste de China (30,3%, 10/33). Un residente chino necesitó trabajar 5,3 días para poder pagar una terapia de inducción de 4 semanas contra la leucemia linfoblástica aguda, el cáncer infantil más frecuente.

**Conclusión:**

El acceso de los niños a los medicamentos esenciales contra el cáncer sigue siendo muy limitado en China y varía de una región a otra. Menos de la mitad de los medicamentos que se estudiaron estaban disponibles y eran asequibles. Se requieren medidas para resolver las posibles carencias y reducir la carga financiera de las familias.

## ملخص

**الغرض:**

التحقيق في إمكانية الوصول إلى الأدوية الأساسية المضادة لسرطان الأطفال في جميع أنحاء الصين.

**الطريقة:**

حصلنا على بيانات لاستخدام الأدوية متعددة القطاعات لعام 2021 من 55 من المستشفيات الثالثة للأطفال في سبع مناطق جغرافية (ثلث مستشفيات الأطفال العامة في البر الرئيسي للصين). تم تقييم معقولية الأسعار من خلال مقارنة الدفع المشترك ليوم واحد لكل دواء بنفس الاسم العام وطريقة التعاطي (أي المنتج)، أو لبرنامج علاج مع الدخل اليومي المنفق للفرد في كل منطقة. تم حساب متوسط توافر، ومعقولية أسعار، جميع أدوية السرطان البالغ عددها 33 دواءً لعام 2021 في القائمة النموذجية لمنظمة الصحة العالمية للأدوية الأساسية للأطفال، ومقارنتها حسب المنطقة، ونوع الدواء.

**النتائج:**

على الرغم من أن كل الأدوية قد تمت الموافقة عليها في الصين، إلا أن 14 (%42.4) منها كانت متاحة في أقل من %50 من المستشفيات، وكانت ستة منتجات (%18.2) منها، يبلغ متوسط الدفع المشترك ليوم واحد لها، يتجاوز الدخل اليومي المنفق. كان متوسط التوافر أعلى بين 19 دواءً تمت الموافقة عليها للاستخدام بواسطة الأطفال، مقارنة بـ 14 دواءً لم تتم الموافقة عليها (%80.0 مقابل %48.2 على التوالي؛ نسبة الاحتمال أقل من 0.001). وبشكل عام، كان %42.4 (14/33) من الأدوية تتمتع بكل من التوافر جيد والأسعار المعقولة؛ وكانت أدنى نسبة في شمال غرب الصين (%30.3، 10/33). واحتاج المواطن الصيني أن يعمل لمدة 5.3 يومًا لتوفير تكاليف العلاج التحفيزي لمدة 4 أسابيع لسرطان الدم الليمفاوي الحاد، وهو أكثر أنواع السرطان شيوعًا بين الأطفال.

**الاستنتاج:**

ظل الوصول إلى الأدوية الأساسية المضادة للسرطان للأطفال دون المستوى الأمثل في الصين، وتفاوت ذلك الوصول عبر المناطق. وكان أقل من نصف الأدوية التي تمت دراستها تتمتع بكل من التوافر الجيد والأسعار المعقولة. وهناك حاجة إلى اتخاذ إجراءات لمعالجة حالات النقص المحتمل، وتقليل العبء المالي على الأسر.

## 摘要

目的 旨在调查中国各地儿童用基本抗癌药物的可及性。

**方法:**

我们从 7 个地理区域的 55 家三级儿童医院（占中国大陆公立儿童医院的三分之一）获得了 2021 年的横截面药物使用数据。通过将具有相同通用名和给药途径的每种药物（即产品）或单个疗程的单日共付额与每个区域的人均每日可支配收入进行比较，我们评估了可负担性。按区域和药物类型计算并比较了 2021 年世卫组织《儿童基本药物标准清单》中列出的所有抗癌药物（共计 33 种）的可获得性和可负担性中位数。

**结果:**

尽管所有药物在中国均已获得批准，但有 14 种（占 42.4%）药物仅在不到 50% 的医院可用，且有 6 种（占 18.2%）产品的单日共付额中位数超过了每日可支配收入。19 种获批用于儿科适应症的药物的可获得性中位数高于 14 种未获批的药物（分别为 80.0% 和 48.2%；*P* <0.001）。总体而言，42.4% (14/33) 药物的可获得性和可负担性均表现良好；中国西北地区的该比例最低（30.3%，10/33）。一名中国居民需要工作 5.3 天，才能负担得起为期 4 周的急性淋巴细胞白血病（最常见的儿童癌症）诱导治疗。

**结论:**

中国儿童用基本抗癌药物的可及性仍未达到理想水平，且各地区之间存在差异性。在所研究的药物中，仅有不到一半药物的可获得性和可负担性均表现良好。必须采取措施以解决潜在的药物短缺问题，并减轻家庭经济负担。

## Резюме

**Цель:**

Изучить доступность основных противоопухолевых препаратов для детей по всему Китаю.

**Методы:**

Были получены перекрестные данные об использовании лекарственных средств за 2021 год из 55 детских больниц третичного звена в семи географических регионах (одна треть государственных детских больниц на территории материкового Китая). Доступность оценивали путем сравнения дополнительной оплаты в течение одного дня за каждое лекарственное средство с одинаковым непатентованным названием и способом применения (то есть препарат) или за курс лечения с ежедневным уровнем дохода после уплаты налогов на душу населения в каждом регионе. Были рассчитаны средние показатели доступности и стоимости всех 33 противоопухолевых препаратов, включенных в *Примерный перечень основных лекарственных средств ВОЗ для детей* на 2021 год, и проведено их сравнение по регионам и типам препаратов.

**Результаты:**

Хотя все препараты были одобрены в Китае, 14 (42,4%) из них были доступны менее чем в 50% больниц, а для шести (18,2%) препаратов средняя дополнительная плата в течение одного дня превышала дневной доход после уплаты налогов. Медиана доступности была выше среди 19 препаратов, одобренных для применения у детей, по сравнению с 14 неодобренными (80,0% по сравнению с 48,2% соответственно; *P* < 0,001). В целом 42,4% (14/33) лекарственных средств были одновременно в наличии и по доступной цене; наименьшая доля была отмечена на северо-западе Китая (30,3%, 10/33). Жителю Китая потребовалось отработать 5,3 дня, чтобы оплатить индукционную терапию продолжительностью 4 недели для лечения острого лимфобластного лейкоза, самого распространенного онкологического заболевания у детей.

**Вывод:**

Доступ к основным противоопухолевым препаратам для детей в Китае остается субоптимальным и различается по регионам. Меньше половины изученных лекарственных средств были в наличии и по приемлемой цене. Необходимо принять меры для решения проблемы потенциального дефицита и снижения финансового бремени для семей.

## Introduction

Cancer is a major cause of death among children and its incidence is growing worldwide.^1^^–^^3^ Globally, there are large variations in childhood cancer survival rates, which range from under 30% in many low- and middle-income countries to around 80% in high-income countries, partly due to unequal access to quality care and pharmacotherapy.^3^^–^^5^ As a result, in 2018 the World Health Organization (WHO) established the Global Initiative for Childhood Cancer, which aims to increase cancer survival rates among children worldwide to at least 60% by 2030 and to provide a foundation for national initiatives on improving survival rates.^6^ As access to essential medicines is critical for childhood cancer survival,^7^ WHO has continuously updated its Model list of essential medicines for children every 2 years since 2007 to help national and regional authorities, especially those in resource-constrained settings, select essential medicines with comparable efficacy and safety.^8^ Nonetheless, ensuring the availability and affordability of essential medicines remains a challenge for low- and middle-income countries.^9^^–^^13^

In China, the incidence of cancer among individuals younger than 20 years was reported in 2021 to be increasing at an average annual rate of 1.8%.^14^ In fact, cancer has become one of the top three causes of death among Chinese children, accounting for an estimated 19.0% of deaths in girls and 14.0% in boys in 2016.^15^ In 2023, a systematic review concluded that the availability of essential medicines during the previous decade in China was low and varied across regions.^16^ However, little research has been conducted on access to essential anticancer medicines for children in China, with some reports restricted to only one province.^17^ Moreover, there is a lack of national data. The aim of this study, therefore, was to investigate the national availability and affordability of essential anticancer medicines for children in China.

## Methods

### Data sources

The National Health Commission of China established the National Hospital Drug Use Monitoring System in 2016. This system requires public hospitals to report drug use data every year, including the generic name of each drug, its formulation and cost and the quantity used. For this study, we extracted cross-sectional data on drug use in 2021 for 55 tertiary children's hospitals, which represented one third of all public children's hospitals in mainland China. Hospitals were divided into seven geographical regions: east, south, central, north, north-west, south-west and north-east China (online repository).^18^^,^^19^ The central region comprised Henan, Hubei and Hunan; the south region comprised Guangdong, Guangxi and Hainan; the north region comprised: Beijing, Hebei, Inner Mongolia, Shanxi and Tianjin; the east region comprised Anhui, Fujian, Jiangsu, Jiangxi, Shandong, Shanghai and Zhejiang; the south-west region comprised Chongqing, Guizhou, Sichuan, Tibet and Yunnan; the north-west region comprised Gansu, Ningxia, Qinghai, Shaanxi and Xinjiang; and the north-east region comprised Heilongjiang, Jilin and Liaoning. According to Peking University Institutional Review Board, ethical approval was not required for this study as the database did not contain any information that could identify individuals.

We also collected data from public databases for each geographical region on: (i) health services (e.g. the number of paediatric beds and health professionals); (ii) health expenditure by government, society and individuals; (iii) gross domestic product (GDP); (iv) population size; and (v) the size of the geographical area, from which we calculated population density and the geographical density of health services (online repository).^19^^–^^21^

Currently, there is no national essential medicines list for children in China. Consequently, we investigated all 27 medicines listed in Section 8.2.1 (Cytotoxic medicines) of the 2021 *WHO Model list of essential medicines for children* and all six medicines listed in Section 8.2.2 (Targeted therapies).^22^ Calcium folinate was excluded because it is a supportive-care agent (online repository).^19^

### Data analysis

We measured the availability and affordability of essential anticancer medicines for children in China using a modified version of the method proposed by WHO and Health Action International.^23^ The availability of a specific medicine was quantified as the proportion of hospitals that reported using the medicine.

The affordability of medicines was assessed according to the route of administration. First, the price of anticancer medicines that had the same generic name and same route of administration was calculated for each geographical region in terms of the median cost per milligram or per unit of each medical product. Second, the cost of using a product (i.e. a medicine with the same generic name and same route of administration) for a single day was estimated from the price per milligram or unit and the minimum single-day monotherapy dose for a standard child (i.e. a child weighing 30 kg with a body surface area of 1 m^2^), based on the product label in China at the end of 2021. If the product could be administered only in combination, the minimum single-day dose of the drug as given in combination therapy was used. If no dose recommendation was available for paediatric patients, we estimated the paediatric dose from the minimum dose per unit body weight or per body surface area in adults. If only the total dose for adults was available, the unit dose for paediatric patients was calculated by dividing the total dose by the average adult body surface area of 1.6 m^2^. The single-day copayment for anticancer products listed in the National Drug Reimbursement List was then calculated by multiplying the single-day cost by the national average copayment rate of 0.3.^24^ Affordability was assessed by determining how many days the lowest-paid unskilled government worker needed to work to afford treatment in accordance with the WHO–Health Action International method. In the absence of official wage data for these workers in China, we used disposable income per capita as a proxy, in line with previous research.^25^^,^^26^ We compared the single-day copayment for anticancer products with the per capita daily disposable income in each region based on the 2022 China Statistical Yearbook, and we expressed the single-day copayment in terms of the number of days’ income required by dividing it by per capita daily disposable income.^20^ Additionally, we calculated the number of days’ income required across all income quintile groups, as determined using data from the 2022 China Statistical Yearbook.^20^

To estimate total medicine cost of, and copayment for, a course of induction therapy and early intensification chemotherapy for a standard child with acute lymphoblastic leukaemia – the most common childhood cancer in China^27^ – we used the calculated unit price and the minimum dosage of first-line treatment regimens recommended in the 2021 Chinese Society of Clinical Oncology diagnosis and treatment guidelines.^28^ This treatment regimen comprised induction therapy with vincristine, daunorubicin, asparaginase or pegaspargase, and prednisone or dexamethasone; and intensification therapy with cyclophosphamide, cytarabine and mercaptopurine. Steroids, such as prednisone, were excluded from the cost analysis because they had multiple indications and were generally lower priced. The copayment was again expressed as the number of days’ income required, which was calculated by dividing the total chemotherapy medicine copayment for a course of each regimen by daily disposable income per capita.

We calculated a median and interquartile range (IQR) for the availability and affordability of each essential anticancer medicine for children. We compared the availability and affordability of all medicines studied, by medicine type and geographical region, using the *χ^2^* test and the Wilcoxon rank sum test, respectively. We considered a two-sided *P-*value less than 0.05 significant. All analyses were conducted using Microsoft Excel 2019 (Microsoft Corporation, Redmond, United States of America) and Stata/SE, version 14.0 (StataCorp LLC, College Station, USA).

## Results

### Availability

All 33 anticancer medicines we identified from the 2021 WHO Model list for children had been approved in China by the end of 2020. Of these, 19 were specifically approved for paediatric indications, including 16 cytotoxic medicines and three targeted therapies (online repository).^19^ The median availability of all 33 medicines was 56.4% (IQR: 40.0–81.8); and the median availability of the 19 approved for paediatric indications was significantly higher than that of the 14 without a paediatric indication: 80.0% (IQR: 51.8–84.5) and 48.2% (IQR: 24.1–58.6), respectively (*P* < 0.001). Overall, 57.6% (19/33) of medicines had an availability of 50% or more (Table 1). Four cytotoxic medicines (12.1%) were not reported as being used by any survey hospital: procarbazine, realgar–*Indigo naturalis* formulation, thioguanine and vinblastine.

**Table 1:** Availability of paediatric anticancer medicines,^a^ by geographical region, China, 2021

Generic name for medicine	Proportion of hospitals reporting medicine use, by geographical region,^b^							
	All (*n* = 55)	Central (*n* = 5)	South (*n* = 9)	North (*n* = 7)	East (*n* = 19)	South-west (*n* = 6)	North-west (*n* = 5)	North-east (*n* = 4)
**Cytotoxic medicine, % (no.)**								
Arsenic trioxide	56.4 (31)	60.0 (3)	33.3 (3)	71.4 (5)	47.4 (9)	83.3 (5)	60.0 (3)	75.0 (3)
Asparaginase	40.0 (22)	60.0 (3)	33.3 (3)	42.9 (3)	26.3 (5)	83.3 (5)	40.0 (2)	25.0 (1)
Bleomycin	78.2 (43)	100.0 (5)	77.8 (7)	57.1 (4)	78.9 (15)	100.0 (6)	40.0 (2)	100.0 (4)
Carboplatin	90.9 (50)	80.0 (4)	100.0 (9)	100.0 (7)	89.5 (17)	100.0 (6)	60.0 (3)	100.0 (4)
Cisplatin	92.7 (51)	100.0 (5)	100.0 (9)	100.0 (7)	94.7 (18)	100.0 (6)	80.0 (4)	50.0 (2)
Cyclophosphamide	96.4 (53)	100.0 (5)	100.0 (9)	100.0 (7)	94.7 (18)	100.0 (6)	80.0 (4)	100.0 (4)
Cytarabine	83.6 (46)	80.0 (4)	77.8 (7)	85.7 (6)	84.2 (16)	83.3 (5)	80.0 (4)	100.0 (4)
Dacarbazine	21.8 (12)	60.0 (3)	11.1 (1)	14.3 (1)	21.1 (4)	50.0 (3)	0 (0)	0 (0)
Dactinomycin	80.0 (44)	100.0 (5)	88.9 (8)	71.4 (5)	78.9 (15)	100.0 (6)	60.0 (3)	50.0 (2)
Daunorubicin	76.4 (42)	60.0 (3)	77.8 (7)	85.7 (6)	68.4 (13)	83.3 (5)	80.0 (4)	100.0 (4)
Doxorubicin	81.8 (45)	100.0 (5)	100.0 (9)	71.4 (5)	73.7 (14)	100.0 (6)	40.0 (2)	100.0 (4)
Etoposide	98.2 (54)	100.0 (5)	100.0 (9)	100.0 (7)	100.0 (19)	83.3 (5)	100.0 (5)	100.0 (4)
Fluorouracil	81.8 (45)	80.0 (4)	77.8 (7)	100.0 (7)	89.5 (17)	100.0 (6)	40.0 (2)	50.0 (2)
Hydroxycarbamide	47.3 (26)	80.0 (4)	77.8 (7)	28.6 (2)	47.4 (9)	50.0 (3)	20.0 (1)	0 (0)
Ifosfamide	85.5 (47)	80.0 (4)	100.0 (9)	85.7 (6)	89.5 (17)	83.3 (5)	80.0 (4)	50.0 (2)
Irinotecan	60.0 (33)	80.0 (4)	66.7 (6)	71.4 (5)	68.4 (13)	50.0 (3)	20.0 (1)	25.0 (1)
Mercaptopurine	54.5 (30)	60.0 (3)	55.6 (5)	71.4 (5)	52.6 (10)	66.7 (4)	40.0 (2)	25.0 (1)
Methotrexate	96.4 (53)	100.0 (5)	100.0 (9)	100.0 (7)	94.7 (18)	100.0 (6)	100.0 (5)	75.0 (3)
Oxaliplatin	30.9 (17)	20.0 (1)	33.3 (3)	0 (0)	42.1 (8)	66.7 (4)	0 (0)	25.0 (1)
Paclitaxel	54.5 (30)	40.0 (2)	88.9 (8)	42.9 (3)	42.1 (8)	83.3 (5)	40.0 (2)	50.0 (2)
Pegaspargase	80.0 (44)	80.0 (4)	77.8 (7)	85.7 (6)	84.2 (16)	83.3 (5)	80.0 (4)	50.0 (2)
Procarbazine	0 (0)	0 (0)	0 (0)	0 (0)	0 (0)	0 (0)	0 (0)	0 (0)
Realgar-*Indigo naturalis* formulation	0 (0)	0 (0)	0 (0)	0 (0)	0 (0)	0 (0)	0 (0)	0 (0)
Thioguanine	0 (0)	0 (0)	0 (0)	0 (0)	0 (0)	0 (0)	0 (0)	0 (0)
Vinblastine	0 (0)	0 (0)	0 (0)	0 (0)	0 (0)	0 (0)	0 (0)	0 (0)
Vincristine	83.6 (46)	80.0 (4)	100.0 (9)	85.7 (6)	94.7 (18)	100.0 (6)	40.0 (2)	25.0 (1)
Vinorelbine	43.6 (24)	20.0 (1)	66.7 (6)	57.1 (4)	42.1 (8)	50.0 (3)	20.0 (1)	25.0 (1)
**Targeted therapy, % (no.)**								
All-trans retinoid acid	49.1 (27)	40.0 (2)	55.6 (5)	71.4 (5)	42.1 (8)	66.7 (4)	40.0 (2)	25.0 (1)
Dasatinib	49.1 (27)	40.0 (2)	44.4 (4)	57.1 (4)	52.6 (10)	66.7 (4)	40.0 (2)	25.0 (1)
Everolimus	7.3 (4)	0 (0)	11.1 (1)	0 (0)	5.3 (1)	16.7 (1)	20.0 (1)	0 (0)
Imatinib	49.1 (27)	60.0 (3)	33.3 (3)	57.1 (4)	52.6 (10)	83.3 (5)	0 (0)	50.0 (2)
Nilotinib	12.7 (7)	0 (0)	11.1 (1)	28.6 (2)	15.8 (3)	16.7 (1)	0 (0)	0 (0)
Rituximab	81.8 (45)	60.0 (3)	88.9 (8)	85.7 (6)	94.7 (18)	83.3 (5)	60.0 (3)	50.0 (2)
**Total, median % (IQR)**								
All medicines	56.4 (40.0–81.8)	60.0 (40.0–80.0)	77.8 (33.3–88.9)	71.4 (28.6–85.7)	52.6 (42.1–89.5)	83.3 (50.0–100)	40.0 (20.0–60.0)	50.0 (25.0–75.0)
All cytotoxic medicines	76.4 (41.8–83.6)	80.0 (50.0–90.0)	77.8 (33.3–100.0)	71.4 (35.7–85.7)	68.4 (42.1–89.5)	83.3 (50.0–100.0)	40.0 (20.0–80.0)	50.0 (25.0–87.5)
All targeted therapies	49.1 (21.8–49.1)	40.0 (10.0–55.0)	38.9 (16.7–52.8)	57.1 (35.7–67.9)	47.4 (22.4–52.6)	66.7 (29.2–79.2)	30.0 (5.0–40.0)	25.0 (6.3–43.8)

IQR: interquartile range.

^a^ The paediatric anticancer medicines studied were those listed in the 2021 *World Health Organization Model list of essential medicines for children*.^22^

^b^ Chinese provinces were grouped into seven regions: (i) the central region comprised Henan, Hubei and Hunan; (ii) the south region comprised Guangdong, Guangxi and Hainan; (iii) the north region comprised: Beijing, Hebei, Inner Mongolia, Shanxi and Tianjin; (iv) the east region comprised Anhui, Fujian, Jiangsu, Jiangxi, Shandong, Shanghai and Zhejiang; (v) the south-west region comprised Chongqing, Guizhou, Sichuan, Tibet and Yunnan; (vi) the north-west region comprised Gansu, Ningxia, Qinghai, Shaanxi and Xinjiang; and (vii) the north-east region comprised Heilongjiang, Jilin and Liaoning.

**Fig. 1:**
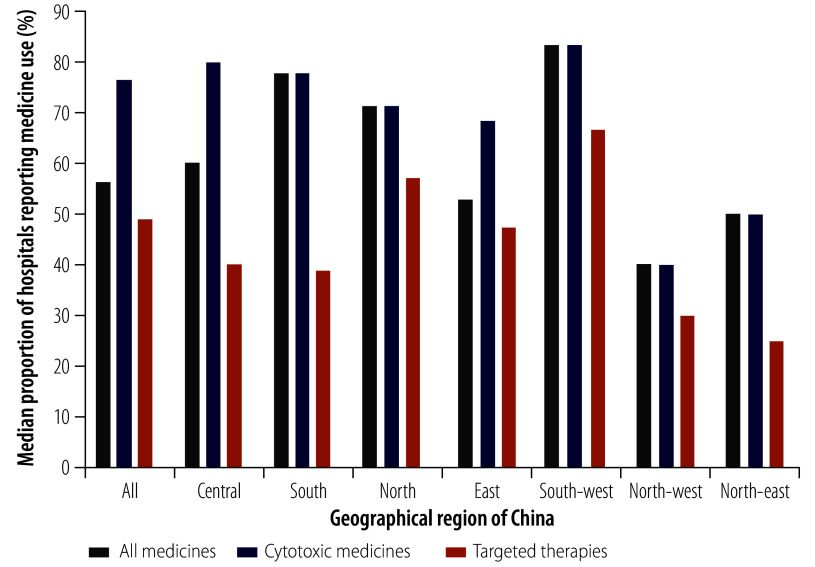
Availability of paediatric anticancer medicines, by geographical region, China, 2021

Notes: The paediatric anticancer medicines studied were those listed in the 2021 *World Health Organization Model list of essential medicines for children*.^22^ Availability was quantified as the proportion of hospitals that reported using the medicine. Chinese provinces were grouped into seven regions: (i) the central region comprised Henan, Hubei and Hunan; (ii) the south region comprised Guangdong, Guangxi and Hainan; (iii) the north region comprised: Beijing, Hebei, Inner Mongolia, Shanxi and Tianjin; (iv) the east region comprised Anhui, Fujian, Jiangsu, Jiangxi, Shandong, Shanghai and Zhejiang; (v) the south-west region comprised Chongqing, Guizhou, Sichuan, Tibet and Yunnan; (vi) the north-west region comprised Gansu, Ningxia, Qinghai, Shaanxi and Xinjiang; and (vii) the north-east region comprised Heilongjiang, Jilin and Liaoning.

The availability of all 33 medicines varied significantly by medicine type and region (Fig. 1). The median availability of cytotoxic medicines was significantly higher than that of targeted therapies: 76.4% (IQR: 41.8–83.6) and 49.1% (IQR: 21.8–49.1), respectively (*P* < 0.001). The lowest availability overall was in north-west (median: 40.0%; IQR: 20.0–60.0) and north-east (median: 50.0%; IQR: 25.0–75.0) China, whereas the highest was in south-west China (median: 83.3%; IQR: 50.0–100). Across regions, the median availability of targeted therapies tended to increase as government and societal health expenditure per 1000 population increased, with the exception of south-west and central China (online repository).^19^

### Affordability

As four cytotoxic medicines were not used by any survey hospital, and four medicines were reported to have two routes of administration (i.e. cyclophosphamide, etoposide, methotrexate and vinorelbine), we estimated the affordability of 33 anticancer medical products with the same generic name and same route of administration, all of which were listed in the National Drug Reimbursement List.^24^ The national median single-day copayment for a standard child ranged from 0.2 yuan (¥; 0.03 United States dollars, US$) for oral hydroxycarbamide to ¥2156.1 (US$ 334.2) for rituximab injections (Table 2; available at: https://www.who.int/publications/journals/bulletin/) the median for all 33 products was ¥19.6 (US$ 3.0). The median single-day copayment for most medicines showed little variation across regions.

**Table 2:** Affordability of paediatric anticancer medicines,^a^ by geographical region, China, 2021

Generic name for medicine (route of administration)	Single-day dose^b^ (mg or unit)	Geographical region^c^																														
		All (*n* = 55)				Central^e^ (*n* = 5)				South (*n* = 9)				North (*n* = 7)				East (*n* = 19)				South-west (*n* = 6)				North-west (*n* = 5)				North-east (*n* = 4)		
		Price per mg or unit,^d^ ¥^e^	Single-day copayment^f^			Price per mg or unit,^d^ ¥^e^	Single-day copayment^f^			Price per mg or unit,^d^ ¥^e^	Single-day copayment^f^			Price per mg or unit^d^, ¥^e^	Single-day copayment^f^			Price per mg or unit,^d^ ¥^e^	Single-day copayment^f^			Price per mg or unit,^d^ ¥^e^	Single-day copayment^f^			Price per mg or unit,^d^ ¥^e^	Single-day copayment^f^			Price per mg or unit,^d^ ¥^e^	Single-day copayment^f^	
			Cost, ¥^e^	No. days’ income required^g,h^			Cost, ¥^e^	No. days’ income required^g,h^			Cost, ¥^e^	No. days’ income required^g,h^			Cost, ¥^e^	No. days’ income required^g,h^			Cost, ¥^e^	No. days’ income required^g,h^			Cost, ¥^e^	No. days’ income required^g,h^			Cost, ¥^e^	No. days’ income required^g,h^			Cost, ¥^e^	No. days’ income required^g,h^
**Cytotoxic medicine**																																
Arsenic trioxide (inj.)	4.8	13.3	19.2	0.2		11.9	17.2	0.2		13.5	19.4	0.2		12.6	18.2	0.2		13.7	19.8	0.2		13.5	19.4	0.3		12.0	17.3	0.2		13.3	19.2	0.2
Asparaginase (inj.)	5000.0	0.0	17.1	0.2		0.0	16.7	0.2		0.0	21.0	0.2		0.0	17.1	0.2		0.0	17.1	0.1		0.0	17.2	0.2		0.0	19.4	0.3		0.0	16.7	0.2
Bleomycin (inj.)	9.4	9.4	26.5	0.3		8.9	25.0	0.3		9.4	26.5	0.2		24.5	69.0	0.7		10.6	29.8	0.3		8.7	24.4	0.3		26.5	74.6	1.0		7.9	22.3	0.3
Carboplatin (inj.)	50.0	0.5	7.8	0.1		0.5	7.8	0.1		0.5	7.8	0.1		0.3	4.0	0.0		0.5	7.8	0.1		0.6	8.3	0.1		0.5	8.2	0.1		0.6	9.1	0.1
Cisplatin (inj.)	15.0	0.8	3.5	0.0		0.8	3.4	0.0		0.9	3.9	0.0		0.8	3.7	0.0		0.7	3.1	0.0		0.6	2.9	0.0		0.9	3.8	0.1		0.8	3.7	0.0
Cyclophosphamide (p.o.)	60.0	0.1	2.0	0.0		NA	NA	NA		NA	NA	NA		NA	NA	NA		0.1	2.0	0.0		NA	NA	NA		NA	NA	NA		NA	NA	NA
Cyclophosphamide (inj.)	90.0	0.1	3.3	0.0		0.1	3.3	0.0		0.1	3.3	0.0		0.1	3.3	0.0		0.1	3.3	0.0		0.1	3.3	0.0		0.1	3.3	0.0		0.1	3.3	0.0
Cytarabine (inj.)	200.0	0.3	16.5	0.2		0.3	16.3	0.2		0.3	16.5	0.2		0.3	16.9	0.2		0.3	16.6	0.1		0.3	16.5	0.2		0.3	16.3	0.2		0.3	20.2	0.2
Dacarbazine (inj.)	75.0	0.6	14.6	0.2		0.5	11.1	0.1		0.4	9.3	0.1		0.8	17.3	0.2		0.6	14.6	0.1		0.6	14.6	0.2		NA	NA	NA		NA	NA	NA
Dactinomycin (inj.)	0.5	518.8	70.0	0.7		518.8	70.0	0.9		518.8	70.0	0.7		518.8	70.0	0.7		518.8	70.0	0.6		518.8	70.0	0.9		518.8	70.0	1.0		518.8	70.0	0.8
Daunorubicin (inj.)	30.0	1.3	12.1	0.1		1.3	12.1	0.1		1.2	10.8	0.1		1.3	12.0	0.1		1.2	11.2	0.1		1.3	11.6	0.2		1.4	12.2	0.2		1.3	12.1	0.1
Doxorubicin (inj.)	36.0	2.2	24.2	0.3		2.3	24.3	0.3		2.2	23.4	0.2		2.3	24.8	0.2		2.2	24.1	0.2		2.2	24.1	0.3		2.3	25.3	0.4		2.2	24.2	0.3
Etoposide (p.o.)	60.0	0.9	16.7	0.2		NA	NA	NA		NA	NA	NA		NA	NA	NA		1.7	29.7	0.3		0.2	3.7	0.0		NA	NA	NA		NA	NA	NA
Etoposide (inj.)	100.0	0.1	2.3	0.0		0.1	2.3	0.0		0.1	2.8	0.0		0.1	2.3	0.0		0.1	2.3	0.0		0.1	3.9	0.1		0.1	2.8	0.0		0.1	2.3	0.0
Fluorouracil (inj.)	300.0	0.2	19.7	0.2		0.2	19.7	0.2		0.2	21.0	0.2		0.2	19.7	0.2		0.2	19.7	0.2		0.2	18.9	0.2		0.2	19.3	0.3		0.2	19.6	0.2
Hydroxycarbamide (p.o.)	600.0	0.0	0.2	0.0		0.0	0.2	0.0		0.0	0.2	0.0		0.0	0.2	0.0		0.0	0.2	0.0		0.0	0.2	0.0		0.0	0.2	0.0		NA	NA	NA
Ifosfamide (inj.)	1200.0	0.1	32.3	0.3		0.1	32.0	0.4		0.1	35.2	0.3		0.1	28.2	0.3		0.2	71.2	0.6		0.1	32.9	0.4		0.1	53.2	0.7		0.1	50.9	0.6
Irinotecan (inj.)	180.0	10.9	589.3	6.1		9.2	497.5	6.2		11.1	601.3	5.6		12.2	660.6	6.5		10.8	583.2	4.9		12.2	660.6	8.6		11.6	629.1	8.8		8.5	457.7	5.4
Mercaptopurine (p.o.)	45.0	0.0	0.4	0.0		0.0	0.5	0.0		0.0	0.5	0.0		0.0	0.4	0.0		0.0	0.4	0.0		0.0	0.5	0.0		0.0	0.5	0.0		0.0	0.3	0.0
Methotrexate (p.o.)	3.1	1.0	0.9	0.0		1.0	0.9	0.0		1.1	1.0	0.0		0.9	0.9	0.0		1.0	0.9	0.0		1.0	0.9	0.0		1.0	0.9	0.0		1.1	1.0	0.0
Methotrexate (inj.)	12.0	0.2	0.8	0.0		2.0	7.0	0.1		0.2	0.6	0.0		1.5	5.3	0.1		0.2	0.6	0.0		0.2	0.7	0.0		1.9	6.9	0.1		2.0	7.1	0.1
Oxaliplatin (inj.)	85.0	1.2	29.6	0.3		0.8	19.6	0.2		0.8	21.2	0.2		NA	NA	NA		1.0	25.9	0.2		3.7	93.8	1.2		NA	NA	NA		3.8	95.7	1.1
Paclitaxel (inj.)	135.0	4.5	183.2	1.9		24.5	990.9	12.3		2.2	89.6	0.8		2.2	89.6	0.9		4.4	179.2	1.5		4.8	194.8	2.5		11.6	468.0	6.5		4.1	164.9	2.0
Pegaspargase (inj.)	2500.0	0.8	596.0	6.2		0.8	595.6	7.4		0.8	596.0	5.5		0.8	596.0	5.9		0.8	596.0	5.0		0.8	596.0	7.8		0.8	596.0	8.3		0.8	596.0	7.1
Vincristine (inj.)	2.0	195.0	117.0	1.2		194.8	116.9	1.5		195.0	117.0	1.1		195.0	117.0	1.2		195.0	117.0	1.0		195.0	117.0	1.5		195.0	117.0	1.6		195.0	117.0	1.4
Vinorelbine (p.o.)	60.0	8.1	145.4	1.5		NA	NA	NA		24.1	433.5	4.0		7.0	126.0	1.2		7.0	126.0	1.1		7.0	126.0	1.6		NA	NA	NA		NA	NA	NA
Vinorelbine (inj.)	25.0	12.7	95.2	1.0		5.4	40.7	0.5		14.1	105.8	1.0		12.0	90.0	0.9		18.6	139.2	1.2		7.5	56.2	0.7		5.5	41.3	0.6		12.7	95.2	1.1
All cytotoxic medicines, median (IQR)	NA	ND	ND	0.2 (0.0–0.5)		ND	ND	0.2 (0.1–0.4)		ND	ND	0.2 (0.0–0.7)		ND	ND	0.2 (0.0–0.7)		ND	ND	0.2 (0.0–0.6)		ND	ND	0.2 (0.0–0.9)		ND	ND	0.3 (0.1–0.9)		ND	ND	0.2 (0.1–1.1)
**Targeted therapy**																																
All-trans retinoid acid (p.o.)	45.0	1.5	19.6	0.2		1.5	19.6	0.2		1.5	19.6	0.2		1.5	19.6	0.2		1.5	19.6	0.2		1.5	19.6	0.3		1.4	19.2	0.3		1.5	19.6	0.2
Dasatinib (p.o.)	62.5	1.3	24.9	0.3		1.3	25.2	0.3		1.4	27.1	0.3		1.4	26.3	0.3		1.2	21.9	0.2		1.3	24.6	0.3		0.6	11.8	0.2		1.4	26.5	0.3
Everolimus (p.o.)	4.5	26.0	35.1	0.4		NA	NA	NA		26.0	35.1	0.3		NA	NA	NA		26.0	35.1	0.3		28.3	38.2	0.5		24.7	33.3	0.5		NA	NA	NA
Imatinib (p.o.)	340.0	0.1	10.1	0.1		0.1	10.6	0.1		0.1	10.0	0.1		0.7	66.4	0.7		0.1	10.6	0.1		0.1	10.0	0.1		NA	NA	NA		0.3	32.6	0.4
Nilotinib (p.o.)	375.0	0.5	55.8	0.6		NA	NA	NA		0.5	55.8	0.5		0.6	64.0	0.6		0.5	53.7	0.5		0.5	55.9	0.7		NA	NA	NA		NA	NA	NA
Rituximab (inj.)	375.0	19.2	2156.1	22.4		19.2	2162.9	26.9		19.3	2167.7	20.1		22.9	2572.2	25.4		15.7	1769.9	15.0		21.0	2365.3	30.8		22.9	8604.2	36.0		46.3	5211.1	61.8
All target therapies, median (IQR)	NA	ND	ND	0.3 (0.2–0.5)		ND	ND	0.3 (0.2–6.9)		ND	ND	0.3 (0.2–0.5)		ND	ND	0.6 (0.3–0.7)		ND	ND	0.2 (0.2–0.4)		ND	ND	0.4 (0.3–0.7)		ND	ND	0.4 (0.2–9.3)		ND	ND	0.4 (0.3–15.7)

inj.: injection; IQR: interquartile range; NA: not applicable; ND: not determined; p.o.: per os (by mouth); ¥: yuan.

^a^ The paediatric anticancer medicines studied were those listed in the 2021 *World Health Organization Model list of essential medicines for children*.^22^

^b^ The single-day dose is the minimum dose of monotherapy given in a single day to a standard child (weight: 30 kg; body surface area: 1 m^2^), as indicated on the product label in China at the end of 2021. If the product could only be administered in combination, the minimum single-day dose of the drug as given in combination therapy was used. If no dose recommendation was available for paediatric patients, we estimated the paediatric dose from the minimum dose per unit body weight or per body surface area in adults. If only the total dose for adults was available, the unit dose for paediatric patients was calculated by dividing the total dose by the average adult body surface area of 1.6 m^2^.

^c^ Chinese provinces were grouped into seven regions: (i) the central region comprised Henan, Hubei and Hunan; (ii) the south region comprised Guangdong, Guangxi and Hainan; (iii) the north region comprised: Beijing, Hebei, Inner Mongolia, Shanxi and Tianjin; (iv) the east region comprised Anhui, Fujian, Jiangsu, Jiangxi, Shandong, Shanghai and Zhejiang; (v) the south-west region comprised Chongqing, Guizhou, Sichuan, Tibet and Yunnan; (vi) the north-west region comprised Gansu, Ningxia, Qinghai, Shaanxi and Xinjiang; and (vii) the north-east region comprised Heilongjiang, Jilin and Liaoning.

^d^ The price was the median cost in yuan (¥) per milligram or per unit of medical products with the same generic name and route of administration, for each region.

^e^ In 2021, ¥ 1 was equivalent to 0.155 United States dollars, according to the State Administration of Foreign Exchange of China.^29^

^f^ The copayment was calculated using the national average copayment rate of 0.3.

^g^ The number of days’ income a resident would need to afford the copayment for a single day of treatment, based on daily disposable income per capita in each region.

^h^ All figures reported are for the absolute number days’ income required except where otherwise indicated.

**Fig. 2:**
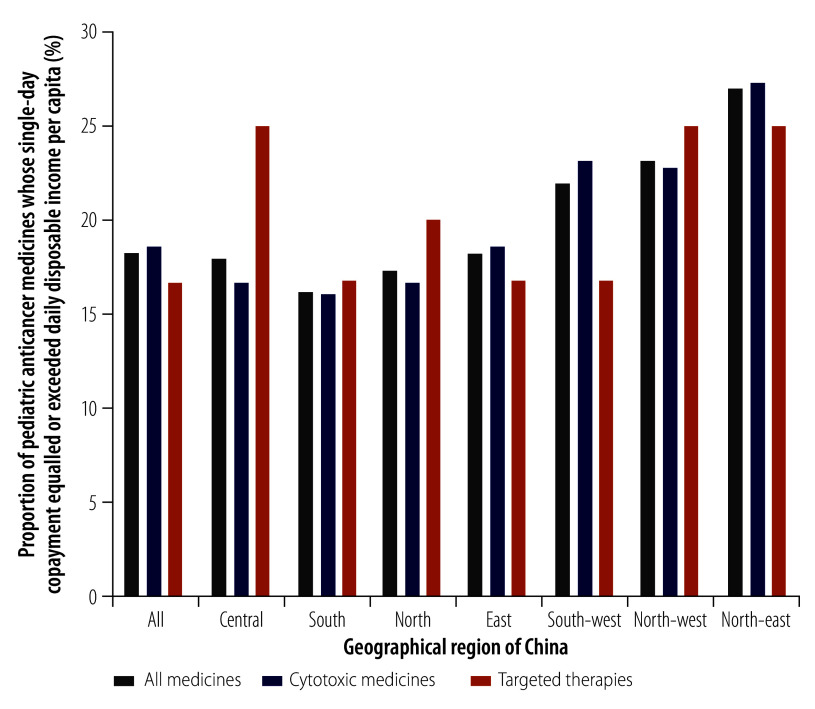
Proportion of paediatric anticancer medicines whose single-day copayment equalled or exceeded daily disposable income per capita, by geographical region, China, 2021

Notes: The paediatric anticancer medicines studied were those listed in the 2021 *World Health Organization Model list of essential medicines for children*.^22^ The graph shows the proportion of medicines studied that had a copayment for a single day of treatment (calculated assuming a national average copayment rate of 0.3) that equalled or exceeded daily disposable income per capita in the region. Chinese provinces were grouped into seven regions: (i) the central region comprised Henan, Hubei and Hunan; (ii) the south region comprised Guangdong, Guangxi and Hainan; (iii) the north region comprised: Beijing, Hebei, Inner Mongolia, Shanxi and Tianjin; (iv) the east region comprised Anhui, Fujian, Jiangsu, Jiangxi, Shandong, Shanghai and Zhejiang; (v) the south-west region comprised Chongqing, Guizhou, Sichuan, Tibet and Yunnan; (vi) the north-west region comprised Gansu, Ningxia, Qinghai, Shaanxi and Xinjiang; and (vii) the north-east region comprised Heilongjiang, Jilin and Liaoning.

For six products (18.2%), the national median single-day copayment equalled or exceeded the daily disposable income per capita (Table 2). In particular, for three products, the copayment was more than five times the daily disposable income per capita for a single-day dose: rituximab injections (22.4 times), pegaspargase injections (6.2 times) and irinotecan injections (6.1 times). People in low-income and lower-middle-income groups needed 94.4 days’ and 42.7 days’ income, respectively, to afford rituximab injections for a single day (Table 3). As shown in Fig. 2, the proportion of medicines whose single-day copayment equalled or exceeded the daily disposable income per capita was highest in north-east China (26.9%; 7/26) and lowest in south China (16.1%; 5/31). Affordability was worse in regions where the geographical density of health services was low. In addition, the median number of days’ income needed for copayments tended to decrease across regions as government and societal health expenditure per 1000 population and gross domestic product (GDP) per capita increased (online repository).^19^

**Table 3:** Affordability of paediatric anticancer medicines,^a^ by income quintile, China, 2021

Generic name for medicine (route of administration)	No. days’ income required to pay for a single-day dose, by income quintile^b,c,d^				
	Low	Lower middle	Middle	Upper middle	High
**Cytotoxic medicine**					
Arsenic trioxide (inj.)	0.8	0.4	0.2	0.2	0.1
Asparaginase (inj.)	0.8	0.3	0.2	0.1	0.1
Bleomycin (inj.)	1.2	0.5	0.3	0.2	0.1
Carboplatin (inj.)	0.3	0.2	0.1	0.1	0.0
Cisplatin (inj.)	0.2	0.1	0.0	0.0	0.0
Cyclophosphamide (p.o.)	0.1	0.0	0.0	0.0	0.0
Cyclophosphamide (inj.)	0.1	0.1	0.0	0.0	0.0
Cytarabine (inj.)	0.7	0.3	0.2	0.1	0.1
Dacarbazine (inj.)	0.6	0.3	0.2	0.1	0.1
Dactinomycin (inj.)	3.1	1.4	0.9	0.6	0.3
Daunorubicin (inj.)	0.5	0.2	0.2	0.1	0.1
Doxorubicin (inj.)	1.1	0.5	0.3	0.2	0.1
Etoposide (p.o.)	0.7	0.3	0.2	0.1	0.1
Etoposide (inj.)	0.1	0.0	0.0	0.0	0.0
Fluorouracil (inj.)	0.9	0.4	0.2	0.2	0.1
Hydroxycarbamide (p.o.)	0.0	0.0	0.0	0.0	0.0
Ifosfamide (inj.)	1.4	0.6	0.4	0.3	0.1
Irinotecan (inj.)	25.8	11.7​	7.4	4.8	2.5
Mercaptopurine (p.o.)	0.0	0.0	0.0	0.0	0.0
Methotrexate (p.o.)	0.0	0.0	0.0	0.0	0.0
Methotrexate (inj.)	0.0	0.0	0.0	0.0	0.0
Oxaliplatin (inj.)	1.3	0.6	0.4	0.2	0.1
Paclitaxel (inj.)	8.0	3.6	2.3	1.5	0.8
Pegaspargase (inj.)	26.1	11.8​	7.5	4.8	2.5
Vincristine (inj.)	5.1	2.3	1.5	1.0	0.5
Vinorelbine (p.o.)	6.4	2.9	1.8	1.2	0.6
Vinorelbine (inj.)	4.2	1.9	1.2	0.8	0.4
All cytotoxic medicines, median (IQR)	0.8 (0.1–2.2)	0.3 (0.1–1.0)	0.2 (0.0–0.6)	0.1 (0.0–0.4)	0.1 (0.0–0.2)
**Targeted therapy**					
All-trans retinoid acid (p.o.)	0.9	0.4	0.2	0.2	0.1
Dasatinib (p.o.)	1.1	0.5	0.3	0.2	0.1
Everolimus (p.o.)	1.5	0.7	0.4	0.3	0.1
Imatinib (p.o.)	0.4	0.2	0.1	0.1	0.0
Nilotinib (p.o.)	2.4	1.1	0.7	0.5	0.2
Rituximab (inj.)	94.4	42.7	27.1	17.5​	9.2
All target therapies, median (IQR)	1.3 (0.9–2.2)	0.6 (0.4–1.0)	0.4 (0.3–0.6)	0.2 (0.2–0.4)	0.1 (0.1–0.2)

inj.: injection; IQR: interquartile range; p.o.: per os (by mouth).

^a^ The paediatric anticancer medicines studied were those listed in the 2021 *World Health Organization Model list of essential medicines for children*.^22^

^b^ The number of days’ income a resident would need to pay for a single day of treatment, based on daily disposable income per capita and assuming a national average copayment rate of 0.3.

^c^ All figures reported are for the number of days’ income required except where otherwise indicated.

^d^ The population was divided into five income groups with approximately 20% in each group, in accordance with the 2022 China Statistical Yearbook.^20^

**Fig. 3:**
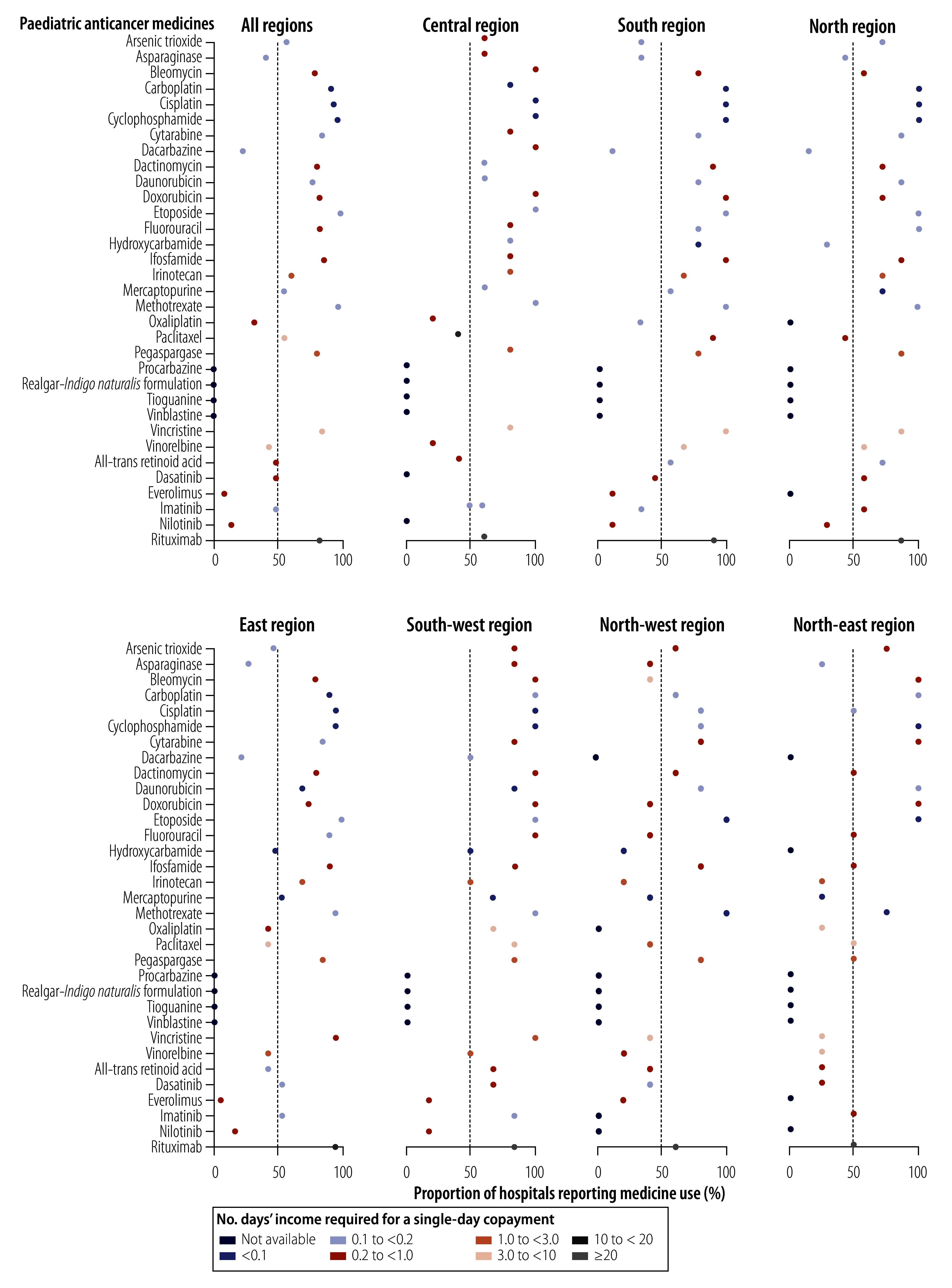
Availability and affordability of paediatric anticancer medicines, by geographical region, China, 2021

Notes: The paediatric anticancer medicines studied were those listed in the 2021 *World Health Organization Model list of essential medicines for children*.^22^ Medicines shown in bold were those approved for paediatric indications in China by the end of 2020. The copayment for a single day of treatment was calculated assuming a national average copayment rate of 0.3 and compared with the daily disposable income per capita in each region. Copayments for products with the same generic name but different routes of administration were averaged. Availability ≥ 50% was regarded as good. A medicine was regarded as affordable if the number of days’ income required to pay for a single day of treatment was less than 1. Chinese provinces were grouped into seven regions: (i) the central region comprised Henan, Hubei and Hunan; (ii) the south region comprised Guangdong, Guangxi and Hainan; (iii) the north region comprised: Beijing, Hebei, Inner Mongolia, Shanxi and Tianjin; (iv) the east region comprised Anhui, Fujian, Jiangsu, Jiangxi, Shandong, Shanghai and Zhejiang; (v) the south-west region comprised Chongqing, Guizhou, Sichuan, Tibet and Yunnan; (vi) the north-west region comprised Gansu, Ningxia, Qinghai, Shaanxi and Xinjiang; and (vii) the north-east region comprised Heilongjiang, Jilin and Liaoning.

Fig. 3 shows the national and regional availability and affordability of each medicine, with the affordability of medicines with the same generic name but different routes of administration being averaged. Overall, 14 medicines (42.4%) were both reported to be used in half or more of the sample hospitals and cost less than the daily disposable income per capita for a single-day dose – all 14 were cytotoxic medicines. The proportion of medicines showing both good availability and good affordability was lowest in north-west (30.3%, 10/33) and north-east (42.4%, 14/33) China.

### Acute lymphoblastic leukaemia

**Fig. 4:**
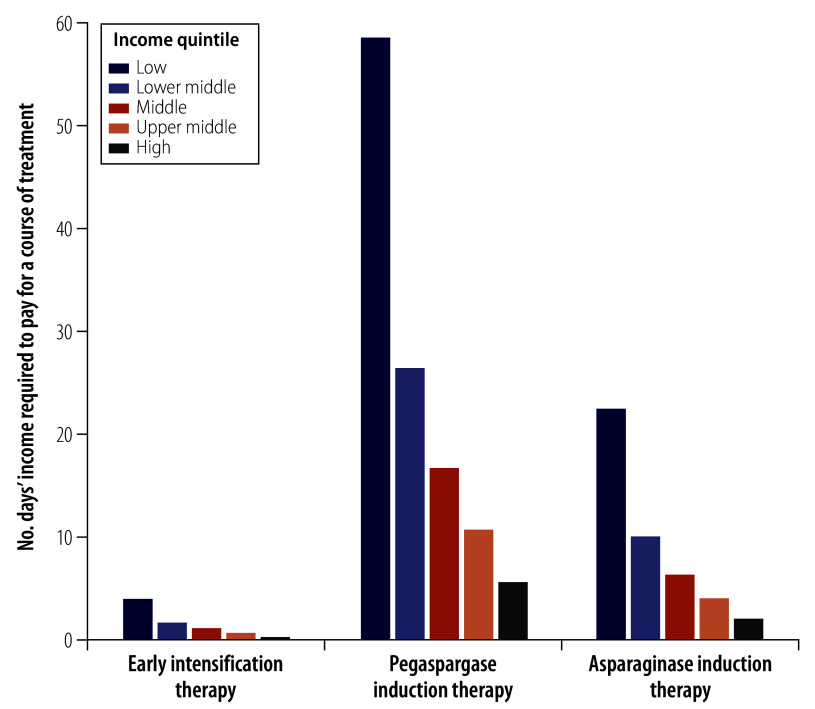
Affordability of chemotherapy medicines for paediatric acute lymphoblastic leukaemia, by income quintile, China, 2021

Notes: Early intensification therapy comprised cyclophosphamide, cytarabine and mercaptopurine for 2 weeks and induction therapy comprised vincristine, daunorubicin, asparaginase or pegaspargase, and prednisone or dexamethasone for 4 weeks. The cost of steroids was not included. The cost of a course of treatment is expressed in terms of the median daily disposable income per capita, assuming a copayment rate of 0.3. The population was divided into five income groups with approximately 20% in each group, in accordance with the 2022 China Statistical Yearbook.^20^

**Fig. 5:**
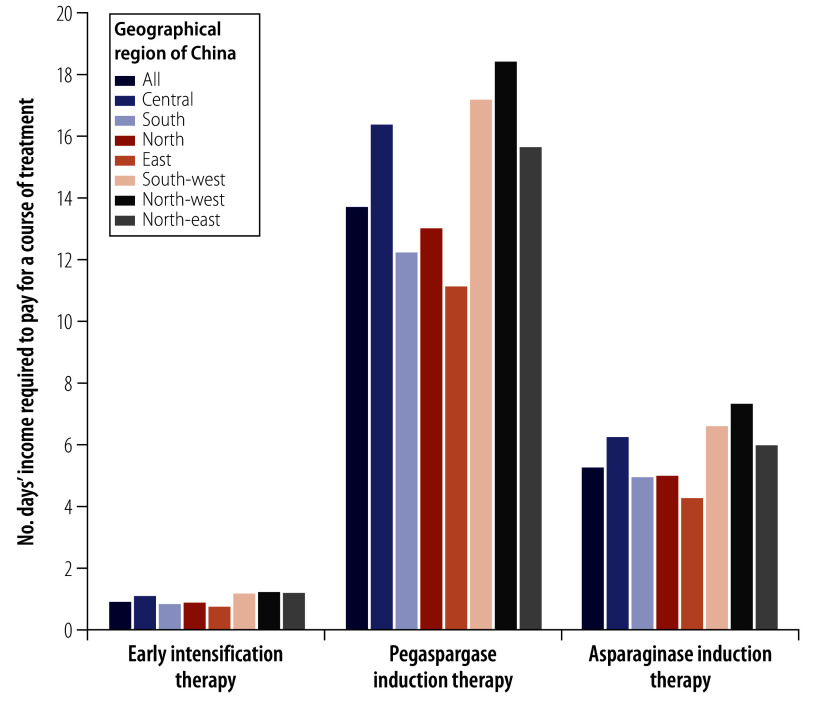
Affordability of chemotherapy medicines for paediatric acute lymphoblastic leukaemia, by geographical region, China, 2021

Notes: Early intensification therapy comprised cyclophosphamide, cytarabine and mercaptopurine for 2 weeks and induction therapy comprised vincristine, daunorubicin, asparaginase or pegaspargase, and prednisone or dexamethasone for 4 weeks. The cost of steroids was not included. The cost of a course of treatment is expressed in terms of the median daily disposable income per capita, assuming a copayment rate of 0.3. Chinese provinces were grouped into seven regions: (i) the central region comprised Henan, Hubei and Hunan; (ii) the south region comprised Guangdong, Guangxi and Hainan; (iii) the north region comprised: Beijing, Hebei, Inner Mongolia, Shanxi and Tianjin; (iv) the east region comprised Anhui, Fujian, Jiangsu, Jiangxi, Shandong, Shanghai and Zhejiang; (v) the south-west region comprised Chongqing, Guizhou, Sichuan, Tibet and Yunnan; (vi) the north-west region comprised Gansu, Ningxia, Qinghai, Shaanxi and Xinjiang; and (vii) the north-east region comprised Heilongjiang, Jilin and Liaoning.

The average Chinese resident needed to work 5.3 days to afford a 4-week course of induction therapy with asparaginase for a standard child with acute lymphoblastic leukaemia, for which the copayment was ¥512.2 (US$ 79.4). If pegaspargase was used instead of asparaginase, the total copayment increased to ¥1329 (US$ 206.0), which was equivalent to 13.8 days’ income (online repository).^19^ For early intensification therapy, the average Chinese resident needed to work 1.0 day to cover the cost of the copayment for a 2-week course, which was ¥92.6 (US$ 14.4). A low-income individual needed to work 22.4 days, and a high-income individual needed to work 2.2 days to afford a course of asparaginase induction therapy (Fig. 4; online repository).^19^ As shown in Fig. 5, the affordability of chemotherapy medicines for acute lymphoblastic leukaemia was least in north-west and south-west China, where 7.4 days’ and 6.7 days’ income, respectively, was required to afford a course of asparaginase induction therapy. If pegaspargase was used instead, 18.5 days’ and 17.3 days’ income would be needed in these two regions, respectively.

## Discussion

Our study revealed that the availability and affordability of essential anticancer medicines for children listed in the 2021 WHO Model list for children at paediatric hospitals in China remained far from ideal. Moreover, access to these medicines varied greatly across geographical regions.

However, we found greater availability than a recent study in Sichuan Province,^17^ which reported that the average availability of generic and branded drugs was 18.5% and 2.6%, respectively, probably because we calculated the availability of all medicines with the same generic name. As anticancer medicines are found in a diverse ranges of dosages, forms and strengths in China,^30^ we would have underestimated availability had we restricted our study to medicines with the specific dosage, form and strength recommended in the WHO Model list.^31^ Nevertheless, our findings show that availability remained suboptimal. The availability of these paediatric medicines in China was similar to that reported in India in 2019 (i.e. about 43% in public hospitals),^11^ which was high for low- and middle-income countries yet remained below that in high-income countries, which have proportions over 80%.^9^^,^^10^^,^^32^

The variations in survival rates and other outcomes in children with cancer observed across countries and regions are almost certainly influenced by inequalities in the availability of medicines and in the quality of health-care services. For example, in 2022, the 5-year net survival rate of children with leukaemia younger than 15 years was 56.3% in China and 47.8% in India, whereas it exceeded 85% in most high-income countries.^4^ Further efforts are needed to increase the availability of essential anticancer medicines for children in low- and middle-income countries. In our study, access seemed to be better for medicines approved for paediatric indications and the availability of cytotoxic medicines was greater than that of targeted therapies. Promoting the timely review and approval of paediatric indications for anticancer medicines may help improve their availability in children’s hospitals. Moreover, no national essential medicines list for children currently exists in China. In low- and middle-income countries, such as Jamaica and Kenya,^10^^,^^13^ expanding the national essential medicines list to include essential cytotoxic medicines for children was one of the enablers of improved access. Consequently, establishing a national list and selecting the appropriate essential medicines for children are crucial for increasing procurement and usage in children’s hospitals. In line with the selection process for WHO Model list for children,^8^ a review committee should be established in China to evaluate which medicines should be included in a national essential medicines list for children.

The affordability of essential anticancer medicines for children remains challenging. As anticancer pharmacotherapy is usually given in cycles over a lengthy period, total medicine costs can be high.^33^^,^^34^ Previous studies in low- and middle-income countries confirm that the cost of anticancer medicines for children are still a substantial burden. For instance, in India 88 days’ income was needed in 2019 to afford the generic medicines to treat a child with acute lymphoblastic leukaemia.^11^ Moreover, a worldwide survey indicated that the cost of cancer treatment in high-income countries was much higher than in low- and middle-income countries after adjustment for exchange rates and purchasing power parity.^32^ Consequently, the affordability of anticancer pharmacotherapy for children remains a global issue.

To make medicines more affordable and to alleviate the financial burden on patients, China has been building a universal basic medical insurance system since the 2009 health system reforms; since 2015, the insurance system has covered over 95% of the population.^35^ The reimbursement rate varies across provinces, insurance schemes, types of health-care visit and hospital levels, ranging from about 50% to 80%.^36^ To control rising medicine costs, the government has engaged in national price negotiations to ensure that expensive medicines are covered by insurance schemes, and has centralized the procurement of anticancer medicines in the medical insurance catalogue.^37^^,^^38^ Nevertheless, although the medical insurance system has greatly decreased costs through reimbursement and price-setting, some anticancer medicines remain unaffordable for many families.^17^ In our study, which used an average copayment rate of 0.3, the estimated copayment for a single day’s treatment with certain medicines was still several times average daily income, particularly for low- and middle-income families. To reduce prices, China includes health technology assessments as part of national price negotiations for anticancer medicines. However, there is some concern that the negotiated price does not always correlate with clinical benefits.^37^ More transparent and credible medical insurance price negotiations for medicines are needed to further improve affordability. In addition, earmarking public funding specifically for essential anticancer medicines for children could help reduce the financial burden on families in low- and middle-income countries.^10^^,^^39^

The inequalities in access to essential anticancer medicines for children we observed across regions could be explained by regional imbalances in economic development and in the allocation of health-care resources. We found that access improved as the geographical density of health services, public health expenditure and GDP per capita increased. Disposable income and GDP per capita are higher in economically developed regions, such as east China, and government and society in these regions spend more on health care per resident than western regions and north-east China.^20^^,^^21^ In addition, large inequalities in the geographical distribution of paediatric anticancer care services still exist in the country, with high-quality services also being concentrated in economically developed regions.^27^ As early diagnosis and timely treatment can reduce the risk of childhood cancer and mortality, inequalities in access to high-quality health care may exacerbate differences in childhood cancer overall survival rates across regions.^27^ Today, however, inequalities in access to health-care services across regions do not occur only in China, they remain a global issue.^40^^,^^41^ Greater efforts are needed to secure pharmaceutical supplies and to improve financial subsidy schemes for underdeveloped regions to promote equal access to essential anticancer medicines for children. This could involve promoting aligned governance, enhancing technical capacities and increasing financial support at the national level.^13^

Our study has several limitations. First, it was based on cross-sectional data on drug use, rather than data on drug procurement or provision. As availability was determined solely from the proportion of hospitals that reported using medicines, we were unable to quantify medicine shortages or trends in access over time.^11^^,^^32^ However, although the lack of data on shortages may have led us to overestimate availability, our findings still reveal that access to essential paediatric anticancer medicines in China is challenging. Second, we studied only tertiary hospitals, which may also have led to overestimates of availability. However, Chinese parents generally bypass primary and secondary health-care facilities for major diseases like cancer and go directly to tertiary hospitals.^42^ Third, we did not differentiate between generic and brand-name drugs, and did not consider whether the dosage forms and strengths were appropriate for children. However, China’s national price negotiations and centralized procurement policy have contributed to price stability for medicines covered by medical insurance.^38^^,^^43^^,^^44^ Fourth, we did not calculate the median price ratio (i.e. the ratio of the local median unit price of a medicine divided by its median international reference price), which is the most common indicator used in price surveys of essential anticancer medicines. International reference prices are usually provided by the Management Sciences for Health’s International Medical Products Price Guide, but the Guide was last updated in 2015 and does not include five newly-added essential anticancer medicines for children.^45^ Instead, to evaluate affordability we compared the single-day copayment for each medicine with daily income per capita, as did previous research.^30^ In addition, because anticancer medicines are usually given in combination, we also estimated the affordability of treatment regimens for acute lymphoblastic leukaemia, which is a practical measure of obvious meaning to caregivers.^11^^,^^32^ Fifth, affordability estimates were based on payments for the minimum dose for a standard child (weight: 30 kg; body surface area: 1 m^2^) as indicated by product labels and guidelines, which may not have been the dose used in complex clinical practice. Sixth, as reimbursement policies were inconsistent across regions and varied widely, we used the national average copayment rate for all regions. Seventh, we were not able to identify associations between medicine access and health outcomes (e.g. survival) due to a lack of data. Finally, our analyses of availability and affordability by region and medicine type were mainly descriptive. Further quantitative and qualitative research is needed to explore determinants of access to essential anticancer medicines for children.

In conclusion, challenges remain in ensuring the availability and affordability of essential anticancer medicines for children in mainland China, both of which could contribute to disparities in childhood cancer survival. Further actions are warranted to address medicines shortages and to alleviate the current financial burden placed on families who require anticancer pharmacotherapy for their children.
